# Complete Amputation of Two Limbs

**Published:** 1902-06-28

**Authors:** 


### COMPLETE AMPUTATION OF TWO LIMBS.

Dr. P. A. Pearcey,[1] of Monkwearmouth, records a case which is extraordinary and probably unique in regard to the proportion of the body successfully removed by primary amputation, the right arm having been amputated at the shoulder-joint and the right thigh at the hip-joint. The patient was a girl aged 17 years, who had been caught between cog-wheels and whirled round by machinery at some brickworks. Her right arm and right thigh were severely smashed and mutilated, but there was little or no hæmorrhage—in fact, the limbs were merely hanging by small skin-attachments. She was suffering from great shock but was conscious. Amputation at the shoulder-joint was first performed and then amputation at the hip-joint. In both cases there was considerable difficulty in obtaining flaps, and before commencing the amputations Dr. Pearcey ligatured the axillary and the femoral arteries respectively. Chloroform was first administered, but this was soon changed to ether. Strychnine was injected subcutaneously, and while the amputation at the hip-joint was proceeding saline solution was infused. The injured parts had been rendered very foul by contact with clay and earth, and extensive cleansing of the tissues was required, so that primary union was not expected. Nevertheless, the shoulder wound healed practically by first intention. The hip, however, gave more trouble, suppurating and healing slowly.

[1] Lancet, June 21.

